# Somatic Variations in Cervical Cancers in Indian Patients

**DOI:** 10.1371/journal.pone.0165878

**Published:** 2016-11-09

**Authors:** Poulami Das, Akanksha Bansal, Sudha Narayan Rao, Kedar Deodhar, Umesh Mahantshetty, Shyam K. Shrivastava, Karthikeyan Sivaraman, Rita Mulherkar

**Affiliations:** 1 Mulherkar Lab, ACTREC, Tata Memorial Centre, Kharghar, Navi Mumbai, 410210, India; 2 Genotypic Technology Pvt. Ltd., #259, Apoorva 4th Cross, 80 Ft Road, RMV II Stage, Bengaluru, 560094, India; 3 Department of Pathology, Tata Memorial Hospital, Parel, Mumbai, 400012, India; 4 Department of Radiation Oncology, Tata Memorial Hospital, Parel, Mumbai, 400012, India; 5 Dhitiomics Technologies Pvt. Ltd. #2/10, 5th Floor, 80 Feet Road, RMV 2nd Stage, Poojari Layout, Bengaluru, Karnataka, 560094, India; German Cancer Research Center (DKFZ), GERMANY

## Abstract

There are very few reports that describe the mutational landscape of cervical cancer, one of the leading cancers in Indian women. The aim of the present study was to investigate the somatic mutations that occur in cervical cancer. Whole exome sequencing of 10 treatment naïve tumour biopsies with matched blood samples, from a cohort of Indian patients with locally advanced disease, was performed. The data revealed missense mutations across 1282 genes, out of 1831 genes harbouring somatic mutations. These missense mutations (nonsynonymous + stop-gained) when compared with pre-existing mutations in the COSMIC database showed that 272 mutations in 250 genes were already reported although from cancers other than cervical cancer. More than 1000 novel somatic variations were obtained in matched tumour samples. Pathways / genes that are frequently mutated in various other cancers were found to be mutated in cervical cancers. A significant enrichment of somatic mutations in the MAPK pathway was observed, some of which could be potentially targetable. This is the first report of whole exome sequencing of well annotated cervical cancer samples from Indian women and helps identify trends in mutation profiles that are found in an Indian cohort of cervical cancer.

## Introduction

With 122800 new cases and 67400 deaths annually, cervical cancer significantly contributes to the cancer burden in India [1]. Although infection by HPV has been established as a major etiological factor for the genesis of the disease, only a small proportion of HPV infected women develop cervical cancer; [2]. This suggests possible involvement of genetic aberrations in development of cervical cancer. Over the years, studies have described direct role of various genetic alterations in disease development. Somatic mutations in PIK3CA, PTEN, TP53, STK11 and KRAS as well as copy number alterations have been reported [2–5]. In addition, recurrent mutations in EP300, MAPKI, FBXW7, HLAB, NFE2L2 have been reported from a large study from Meyerson’s group [6].

Integration of HPV in the human genome could also contribute to genetic aberrations and alter gene expression, leading to cervical carcinogenesis. We and others have reported that the site of HPV integration is not totally random [7, 8]. Meyerson’s group has reported that gene expression levels at sites of HPV integration were higher in tumours with integration than level of expression in other tumours without integration at that site [6]. HPV integration may result in loss of function of tumour suppressors or critical DNA repair gene or gain of function of important oncogenes [9]. For instance, integration in the DNA repair gene RAD51B results in amplification of an intronic segment and expression of alternative *RAD51B* transcripts which are non-functional [10]. Similarly, integration in the tumor suppressor gene *ETS2* is accompanied by deletion of exons at the integration site, resulting in truncated forms of the protein [10]. The integration process has also been reported to alter the copy number of potential oncogenes such as MYC, FOXE1, etc [9].

In the present study, we report the application of whole-exome sequencing technology for identifying trends in mutation profiles found in an Indian cohort of cervical cancer.

## Materials and Methods

### Clinical Samples

Ten pretreatment cervical tumour biopsies along with the matched blood samples from Indian patients with advanced stage cervical cancer (FIGO stage IIIB) were taken for the study. A generic consent for basic research was obtained prior to obtaining the biopsies. Since this was a retrospective study, patient informed consent for this particular project could not be obtained and permission for waiver from Institute Ethics Committee (IEC) was obtained. The study was approved by IEC. All procedures pertaining to sample collection and processing were in conformity with the IEC. The biopsies were obtained and stored in liquid Nitrogen as described earlier [8], from histologically proven, primary cervical tumour, before the start of radical radiation therapy. The samples were coded for identification by the physician prior to testing and were assigned a laboratory code to maintain confidentiality. Buffy coat was separated from blood samples by centrifugation.

### Processing of tumour samples and isolation of genomic DNA

Cryosectioning of the cervical biopsies was done for hematoxylin-eosin (H&E) staining as well for isolation of DNA. The H&E stained sections were examined by the pathologist to assess the percentage of tumour in the sample. Samples with >70% tumour were taken for whole exome sequencing study. Genomic DNA was isolated from the tumour sections and buffy coat from matched blood samples, using DNA Mini kit (Qiagen, Hilden, Germany) according to the manufacturer’s instructions. RNase treatment of the DNA samples was done using RNase A (Sigma, St. Louis, MO, USA).

### Exome Capture and Next-Generation Sequencing

Matched tumour and blood samples from 10 cervical cancer patients were subjected to exome sequencing using Illumina platform. Exome capture and paired end sequencing of DNA was done using Paired-End Sample Preparation kit (Illumina, San Diego, CA, USA) followed by in-solution capture of genomic DNA using SureSelect Human All Exon kit (Agilent Technologies, Santa Clara CA, USA), targeting 37 Mb sequence from exons. For each sample, captured DNA was sequenced by multiplexed paired end sequencing using 2 pools of samples on Illumina GAIIx platform (Illumina, San Diego, CA, USA)

### Bioinformatic analysis outline

The data were QC filtered and mapped to Human Genome version 37 (hg37.p13) using Bowtie. Variants were called using *lofreq* [11], which calls SNVs from each dataset (normal and tumour) and identifies somatic variations–including low frequency, rare alleles. The SNVs called by *lofreq* were subjected to quality filtering based on a) read depth for each variation; b) strand bias; c) end mapping bias; d) mapping quality; e) base RMS quality; and f) variant phred score. A given SNV was considered to be novel if it was not in the dbSNP nor in the 1000 genome database. The resultant variations were submitted to Variant Effect Predictor server (http://www.ensembl.org/info/docs/tools/vep/index.html) and the effect of coding site variants identified. Further analyses were performed for the variations that resulted in a missense or nonsense mutation in coding regions (non-synonymous). The genes containing these SNVs were analyzed for their functional enrichment using GO classification (AMIGO2; http://amigo.geneontology.org). These were also subjected to KEGG pathway analysis (www.genome.jp/kegg/tool/search_pathway.html) and compared to those previously reported in the COSMIC database (cancer.sanger.ac.uk/cancergenome/projects/cosmic/).

### Validation of the data by Sanger sequencing

A subset of variations was validated by PCR amplification of the region followed by Sanger sequencing of the amplicon. Primers were designed flanking the variation ~250 bp upstream and 250 bp downstream using NCBI primer Blast (http://www.ncbi.nlm.nih.gov/tools/primer-blast/). Standard PCR conditions were followed with the annealing temperature in the range between 55–65°C. The PCR products were purified using QIAquick PCR Purification kit (Qiagen, Hilden, Germany) and sequenced on DNA Sequencer (3100 Avant Genetic analyzer, Applied Biosystems, Foster City, CA, USA).

## Results

### Clinical material

In the present study, 10 tumour biopsies along with matched blood from treatment naïve cervical cancers from a cohort of Indian women were included. The patients were in FIGO stage IIIB and their age ranged between 35–58 years. The HPV genotype and viral genome status in these patients was determined from an earlier study [8, 10]. Majority of the samples were positive for HPV16, although HPV18, 45 and 56 were also present [12]. In some of the samples, the virus was integrated in the genome [8]. The demographic details of the patients are given in Table 1.

**Table 1:** Demographic details of the 10 patients included in the whole exome sequencing study

Sample ID	Histology	FIGO stage	Age (yrs.)	Disease status	HPV genotype*	Viral genome status^#^	Integration site^#^
1123	SCC	IIIB	40	NED	16	Integrated	Unknown
999	SCC	IIIB	49	NED	16	Integrated	4q13.3
938	SCC	IIIB	50	LR + DM	16, 18	Not known	
1099	SCC	IIIB	38	NED	16	Not known	
1103	SCC	IIIB	50	DM	16	Not known	
755	SCC	IIIB	44	LR	16	Integrated	3q23
785	SCC	IIIB	43	LR	16, 18	Integrated	3q23, 20q11.21
937	SCC	IIIB	50	LR	Not detected	-	-
1023	SCC	IIIB			16	Not known	
1094	SCC	IIIB	58	DM	16	Integrated	13q

**Key:** SCC- Squamous cell carcinoma; NED-No Evidence of Disease; LR-Local Recurrence; DM-Distant Metastasis; HPV-Human Papilloma Virus

*HPV was determined by Luminex Bead Array and HPV type specific PCR

^#^Viral genome status and integration site were determined by APOT assay as described in Ref. 8

### Exome sequencing and somatic variations

**Fig 1:**
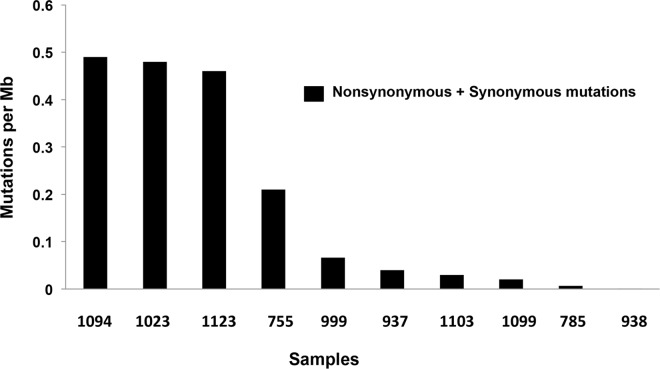
Frequency of somatic mutations in each of the 10 cases. The frequency of all the mutations (synonymous and nonsynonymous) per Mb of genome is depicted. Each bar represents an individual tumour sample and the number below the bar is the designated laboratory number of each sample.

**Fig 2:**
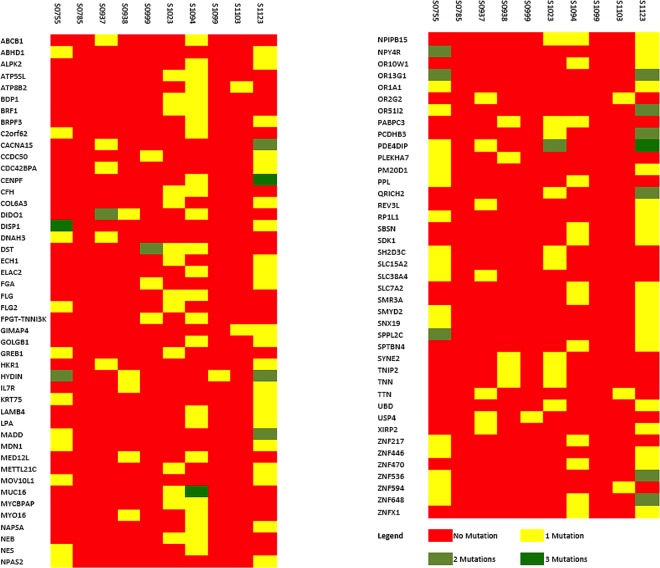
Heat map of frequently mutated genes. A panel of 87 genes was found to be mutated in at least 2 tumour samples. Some of the genes had 3 mutations in the same sample.

The matched tumour and blood samples from 10 cervical cancer patients were subjected to exome sequencing using Illumina platform. On an average 97% of the reads could be mapped to the human genome (hg37.p13) and the average read depth was >20X. The matched blood control in each case was used to filter out the germline variations. Further quality filtering by methods as described in Materials and Methods, identified 2281 somatic variations falling within coding regions of which 1233 (54%) were of missense type, 68 (3%) stop gained, and the rest were synonymous coding and stop (retained) variations. The frequency of mutations in each of the 10 cases is shown in Fig 1. A total of 1831 genes were found to harbour somatic variations. A total of 299 genes carried at least 2 variations (either in same tumour or across tumours). A panel of 87 genes were found to be mutated in at least 2 tumours (Fig 2).

Two genes, PDE4DIP (mutated in 4 tumours) and MUC16 (mutated in 2 tumours) carried 11 somatic variations each, making them the most mutated genes in the dataset. MUC17 showed a high frequency of mutations in a single sample. Apart from this, 80 ZNF family members representing 4.5% of the total number of genes harbouring somatic mutations were also found to be mutated. ZNF proteins had an overrepresentation in the list of somatically mutated genes, when compared to their overall frequency in the human genes (538 out of 32620 genes: 1.6%). This overrepresentation was statistically significant (Fisher’s p = 2.6 x 10–13; OR = 2.72; 95% CI = 2.11–3.47).

MAPK signal transduction pathway that is well known to play a key role in the pathogenesis of cancer, surfaced as one with most mutated genes in this dataset. KEGG lists 257 genes as part of the MAPK signal transduction pathway, including the core signal transduction cascade, the upstream ligands and other associated factors. Twelve of the 257 genes were identified to have somatic variations across patients. In order to assess the significance of this occurrence, we generated 1000 random gene sets of 257 each and calculated the frequency of observed somatic mutations in these sets; Z-scores were used as a measure of significance. The Z-score for finding 12 genes with somatic mutations out of 257 random genes was 2.69 (P-value = 0.004). This indicates a significant enrichment for somatic mutations in the MAPK pathway of these tumour samples

(Fig 3).

**Fig 3:**
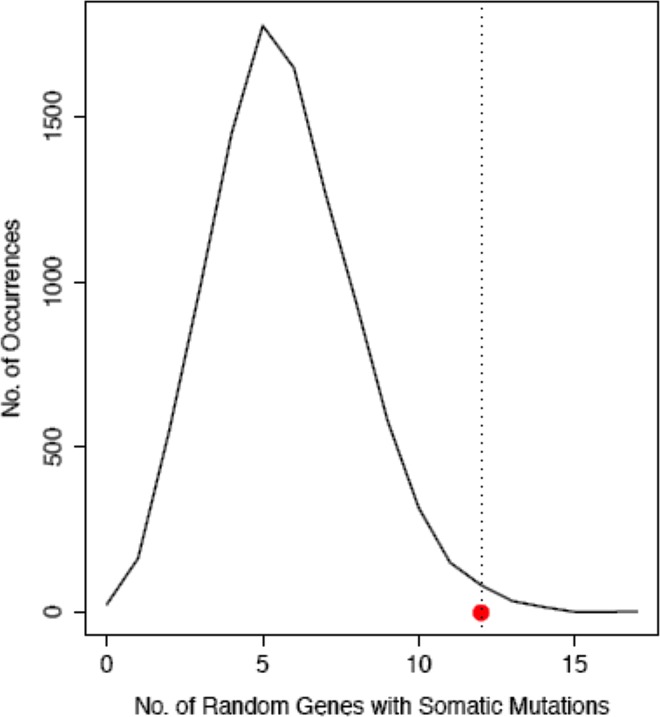
Randomization test for somatic mutations. The curve represents the distribution of number of genes that belonged to the MAPK pathway in the 1000 sets of random genes. The mean of the data set is 5.7 somatic mutations in 257 genes with a standard deviation of 2.34. The red dot along the X-‐axis depicts a value of 12 –the number of MAPK genes having somatic mutations. The P-value was calculated based on the number of random sets that had less than 12 genes belonging to MAPK pathway. The number of somatic mutations observed in MAPK pathway is significantly higher than the mean (P = 0.0046).

Missense somatic variations from each paired tumour sample were used to analyze functional enrichment under the GO molecular function categories. All GO category enrichments with an adjusted p = 0.05 or less were considered to be significant. We did not find any negative enrichment (underrepresentation) of GO functional categories. In a combined GO analysis across multiple samples olfactory receptors, ion binding, signalling cascade receptor and signal transduction genes were found to be enriched.

### Validation of selected variations by Sanger sequencing

A total of 53 somatic variations were randomly selected for variation by Sanger sequencing. Out of this, 25 were validated as true somatic and 10 turned out to be germline (Table 2).

**Table 2:** Variations from exome sequencing data validated by Sanger sequencing.

Sr. No	Gene name	Mutation	Amino acid change	Variation Type	Nature of variation	COSMIC overlap	Importance	Domain
1.	FGF7	tAc/tGc	Y76C	Missense	Somatic	No	MAPKinase signaling pathway; pathways in cancer	FGF domain
2.	TGFBR2	Cag/Tag	Q220*	Stop gain	Somatic	No	MAPKinase pathway; pathways in cancer	
3.	TAF5	Gag/Cag	E383Q	Missense	Somatic	No	Basal transcription factor	
4.	MAP3K3	Gag/Aag	E645K	Missense	Somatic	No	MAPKinase pathway	
5.	DIDO1	tCg/tTg	R303Q	Missense	Somatic	Yes	Involved in apoptosis	PHD Zinc finger domain
6.	DIDO1	ctC/ctG	E301Q	Missense	Somatic	No	Involved in apoptosis	PHD Zinc finger domain
7.	SOS2	tGa/tAa	S1049L	Missense	Somatic	No	MAPKinase pathway; pathways in cancer.	
8.	RASA1	tGg/tAg	W490*	Stop gain	Somatic	No	MAPKinase pathway	PH domain
9.	EP300	Gat/Aat	D1399N	Missense	Somatic	Yes (stomach and cervix)	Signaling pathway; Play a role in epithelial cancer	
10.	RNF111	Cag/Tag	Q605*	Stop gain	Somatic	Yes (acute myeloid leukaemia)	Regulation of transcription	
11.	COL15A1	Gga/Tga	G990*	Stop gain	Somatic	No	Signal transduction; Role in angiogenesis	
12.	NCOR2	aCc/aAc	G1289V	Missense	Somatic	No	Notch signaling pathway	
13.	EDNRB	Caa/Gaa	L233F	Missense	Somatic	No	Regulation of transcription; phospholipase C-activating G-protein coupled receptor signaling pathway; etc.	
14.	MYD88	Gac/Cac	D113H	Missense	Somatic	No	regulation of I-kappaB kinase/NF-kappaB cascade and NF-kappaB transcription factor activity	
15.	CD274	Gaa/Caa	E152Q	Missense	Somatic	No	Cell adhesion molecules (CAMs); signal transduction	
16.	CDCA7L	GTa/gCa	Y64C	Missense	Somatic	No	positive regulation of cell proliferation; regulation of transcription	
17.	PRPF4B	cGt/cAt	R856H	Missense	Somatic	No	protein serine/threonine kinase activity	
18.	ABCG5	cGt/cAt	T489M	Missense	Somatic	No	ATPase activity; ABC transporters	Transmembrane domain
19.	NOL9	acC/acG	G153R	Missense	Somatic	No	polynucleotide 5'-hydroxyl-kinase activity	
20.	NOL9	ctC/ctG	E185Q	Missense	Somatic	No	polynucleotide 5'-hydroxyl-kinase activity	
21.	PTPDC1	Gag/Aag	E639K	Missense	Somatic	No	protein tyrosine/serine/threonine phosphatase activity	
22	ODZ1	ctG/ctA	Q957*	Stop gain	Somatic	No	negative regulation of cell proliferation	
23.	ALS2CR8	Gaa/Aaa	E688K	Missense	Somatic	No	sequence-specific DNA binding transcription factor activity	
24.	MACF1	Cgg/Tgg	R2091W	Missense	Somatic	No	cell cycle arrest	Spectrin domain
25.	TTN	gaC/gaT	V2387I	Missense	Somatic	Yes (breast cancer)	protein serine/threonine kinase activity; protein tyrosine kinase activity	
26.	G6PD	caC/caT	V390M	Missense	Germline	No	Metabolic pathways	
27	FGF22	Cgg/Tgg	R100W	Missense	Germline	No	MAPKinase Pathway; Pathways in cancer	FgGF domain
28.	FMOD	gGa/gAa	S105F	Missense	Germline	No	transforming growth factor beta receptor complex assembly	LRR domain
29.	HIVEP3	ctC/ctT	E155K	Missense	Germline	No	positive regulation of transcription, may be a previously unidentified tumor-suppresser gene	
30.	AIM1	Cat/Aat	H219N	Missense	Germline	No		
31.	SEPN1	Agc/Ggc	S388G	Missense	Germline	No		
32.	SEPN1	Gtg/Atg	V534M	Missense	Germline	No		
33.	HAGH	gaC/gaA	V207F	Missense	Germline	No	Metabolic pathways	
34.	GRAMD1A	cGc/cAg	R117Q	Missense	Germline	No		Gram Domain
35.	GRAMD1A	cCc/cGc	P554R	Missense	Germline	No		Gram Domain

Key:

* indicates termination of the amino acid chain

### Comparison with COSMIC database

From the present dataset missense mutations were catalogued across 1282 genes, out of 1831 genes harbouring somatic mutations. These missense variations (nonsynonymous + stop-gained) were compared with pre-existing mutations in the COSMIC database. 272 mutations in 250 genes were common with those reported in COSMIC database (see S1 Table). However, the data did not match any known cervical cancer mutations.

## Discussion

Although HPV is the major causative factor for development of cervical cancer, the role of several genetic components in the disease has long been recognized. While allelic variation of HLA class II antigen [13, 14], and p53 codon 72 polymorphism [15] may increase the risk of developing the disease, mutations in Ras oncogene, PI3KA, STK11, are reported to directly cause or promote progression of cervical carcinoma [2, 3, 5]. Apart from this, a number of chromosomal aberrations have also been associated with the disease [4]. However, till date there are very few high throughput studies that describe the genetic landscape of the disease. The study by Ojesina et al. involving 100 Norwegian and 15 Mexican cervical carcinoma cases is the most significant among them. They identified recurrent mutations in MAPK, and mutations in genes such as HLA-B, EP300, FBXW7, NFE2L2,TP53 and ERBB2 in 79 primary squamous cell carcinomas and that of ELF3 and CBFB in 24 adenocarcinomas [6]. A study from Latin America reported mutations in PIK3CA to be a key player both in squamous and adeno cervical carcinomas [16]. Another study on cervical cancer cases of European origin identified PIK3CA, KRAS and FBXW7 to be the most mutated [17]. Chung et al have reported mutations in FAT1, ARID1A, ERBB2 and PIK3CA in 15 cervical adenocarcinomas cases of Chinese origin [18]. However, there are no reports on such studies on the Indian population. In the present study, using whole exome sequencing technology we have attempted to catalogue the somatic aberrations in a cohort of Indian cervical cancer cases. Initially identified as an effective tool for identification of a wide variety of novel variations especially in the field of Mendelian disorders [19], over past few years the technology has been quiet successful in discovering novel mutations in diseases characterized by marked genetic heterogeneity like cancer including the rare forms [20, 21].

Whole exome sequencing of 10 paired advanced stage cervical cancer biopsies from Indian cohort identified 1029 novel, non-synonymous variations. High frequency of mutations in two genes—PDE4DIP (a number of mutations in the same gene across samples) and MUC17 (a number of mutations in the same gene in only one sample) were found. Known for its role in anchoring phosphodiesterase 4D to the Golgi/centrosome region of the cell, deleterious mutations of PDE4DIP have recently been demonstrated to be involved in breast cancer [22].

A number of membrane-associated mucin genes such as MUC2, 4, 5B, 12, 16, 17, 20, 21 were found to be mutated in our dataset. Similar to our results, whole exome sequencing study of HPV negative versus HPV positive head and neck tumours identified MUC4, 12 and 16 as mutated [23]. We found MUC16 to be recurrently mutated across the samples. A number of MUC mutations have been reported in the COSMIC database for several cancer types. In general, the membrane associated mucins are also known to play role in invasion and metastasis by modulating the adhesive properties of cells [24]. On the other hand, it is possible that mutations in Muc16 as well as Muc4 are false positives as these are large genes which are low expressed and late replicating and therefore show a high frequency of mutations in the coding region [25].

An interesting finding from the present study was the overrepresentation of mutated members of the ZNF family. A total of 80 ZNF genes were found to be mutated. Zinc Finger members have been implicated in tumorigenesis and acquisition of resistance to drugs. Members of these family interact with nucleic acids, proteins and small molecules and are involved in a variety of crucial molecular processes in cells at replication, transcriptional and translational levels. Mutations of ZNF family members are associated with various diseases including cancer. Zinc Finger genes map to regions often deleted in solid tumours and involved in recurrent chromosomal rearrangements in hematological malignancies [26]. Deleterious mutation of one of the ZNF family members, PRDM1 has been associated with primary central nervous system lymphoma [27]. Corroborating our findings, a recent study has reported multiple mutations of the ZNF family genes in metastatic hepatocellular carcinoma. [28]. As reported in the study by Nicholas et. al., for HPV positive and negative head and neck squamous cancer, ZNF family members including ZNF10, ZNF470 and ZNF716 were also found to be mutated in our dataset [23]

GO analysis identified a large number of olfactory receptor gene mutations. Mutations in olfactory receptors are now being associated with cancer [29] [30], tumour invasion and metastasis [31]. However, according to a recent report, high frequency of somatic mutations in these genes may be red herrings during high throughput sequencing of cancer genomes. According to this study, genes such as the olfactory receptors that have low expression, are late in replicating and display a high regional noncoding mutation rate, are more prone to somatic mutations in their coding regions and hence are seen in many cancer genomics data [25]. Therefore, in order to conclude whether mutations in these receptors play any role in cervical carcinogenesis, more samples need to be sequenced as well as functional analysis of these genes in cell lines and patient samples is required.

Of the mutations that have occurred in single samples and have been identified and validated from the dataset, there were several significant candidates known for their role in the process of tumorigenesis. For instance FGF7 is known for its mitogenic and cell survival activities [32]; MAP3K3 is involved in direct activation of stress-activated protein kinase (SAPK) and extracellular signal-regulated protein kinase (ERK) pathways [33] as well as plays a critical role in TNF-induced NF-kappaB activation [34]. RASA1 helps in control of cellular proliferation and its mutation is associated with basal cell carcinoma [35]. RNF111 acts as a tumour suppressor in preventing development and progression of colorectal cancer [36]. CDCA7L interacts with c-Myc in bringing about cellular transformation in medulloblastoma [37]. Mutation of CD274 leading to its overexpression have been associated with gastric carcinoma [38]. Apart from these, mutations in ODZ1, TTN and EP300 are already reported in cervical cancer. The variation in the transcriptional regulator, EP300 (D1399N) was identical to that reported in COSMIC database. EP300 was also reported to be one of the genes with significant recurrent somatic mutations in the study by Ojesina *et al* [6].

Despite the important role played by each of these genes, majority of the mutations would be ‘passengers’ and identifying potential ‘drivers’ with a cardinal role in cervical carcinogenesis from this data would be the actual challenge. Several methods have been described that can predict potential driver mutations in a cancer genome [39, 40]. These, combined with functional characterization of the variants and validation studies involving larger sample size might help in identifying potential ‘drivers’ in cervical carcinogenesis.

A significant proportion of the genes found to be mutated such as MAP3K3, SOS2, FGF7, TGFBR2, TRAF2, RASA1 and RPS6KA6 belong to the MAPKinase signal transduction pathway that is known to play a significant role in cell proliferation and apoptosis. This observation is in concordance with the study by Ojesina *et al* [6]. This indicates that the MAPKinase pathway is one of the highly mutated pathways in cervical carcinogenesis and hence might be more vulnerable to therapeutic intervention.

The major highlight of our study was to identify non-synonymous, somatic variations (mutations) that might play a potential role in cervical carcinogenesis. We could not find any significant driver mutation that could lead to cervical carcinogenesis. This might be due to the fact that all (except one) of the samples were positive for high risk HPV which are known to target the two key cell cycle regulators-p53 and Rb for proteasomal degradation, without incurring mutation. In our study, HPV infection in the samples and its integration was determined by Luminex Bead Array [8]. Presence of HPV and its integration status was also confirmed in the exome sequencing data of the samples [12]. Thus, our treatment naïve primary cancers could be HPV driven and hence no driver mutations are seen.

Our results suggest that there is considerable genetic and mechanistic heterogeneity in cervical cancers. Pathways / genes that are frequently mutated in various other cancers are also mutated in cervical cancers indicating that specific mutational signatures notwithstanding, distinct cancers based on their tissue of origin, are offshoots of the same mechanistic process. Thus, a tumour driving pathway in prostrate or lung cancer may well be the culprit even in cervical cancer. Given this convergence of pathways / mechanisms across cancer types, one can explore the possibility of cross-treating cancers, for example, the same drug may be effective against two divergent cancer types if they carry the same mutational signature at the pathway level.

To the best of our knowledge, this is the first study where whole exome sequencing has been applied to understanding the mutational landscape of the disease in an Indian cohort. However, there might be several polymorphisms that predispose an individual to the disease or aid the HPV in causing cervical cancer, which we have not dealt with in this study. An additional aspect of the study is that it provides an insight into the genome of Indian individuals by identifying a number of novel variations which could enrich the existing databases.

## Supporting Information

S1 Table List of somatic mutations in common with COSMIC database (XLSX)
